# Selenium levels and their association with thyroid autoimmunity and severe preeclampsia in pregnancy: Insights from a prospective ideal breast milk cohort study

**DOI:** 10.1530/ETJ-24-0007

**Published:** 2024-07-09

**Authors:** Chae Won Chung, Kyungsik Kim, Sue K Park, Dal Lae Ju, Young Joo Park, Choong Ho Shin, Jong Kwan Jun, June-Key Chung, Yoon Ju Song, Young Ah Lee, Gi Jeong Cheon, Sun Wook Cho

**Affiliations:** 1 Department of Internal Medicine, Seoul National University Hospital and Seoul National University College of Medicine, Seoul, Korea; 2 Department of Internal Medicine, Chung-Ang University Hospital, Seoul, Korea; 3 Department of Preventive Medicine, Seoul National University College of Medicine, Seoul, Korea; 4 Cancer Research Institute, Seoul National University College of Medicine, Seoul, Korea; 5 Integrated Major in Innovative Medical Science, Seoul National University College of Medicine, Seoul, Korea; 6 Department of Food Service and Nutrition Care, Seoul National University Hospital, Seoul, Korea; 7 Department of Molecular Medicine and Biopharmaceutical Sciences, Graduate School of Convergence Science and Technology, Seoul National University, Seoul, Korea; 8 Department of Pediatrics, Seoul National University Children’s Hospital and Seoul National University College of Medicine, Seoul, Korea; 9 Department of Obstetrics and Gynecology, Seoul National University Hospital and Seoul National University College of Medicine, Seoul, Korea; 10 Department of Nuclear Medicine, Seoul National University College of Medicine, Seoul, Korea; 11 Department of Food Science and Nutrition, The Catholic University of Korea, Bucheon, Korea

**Keywords:** preeclampsia, selenium deficiency, selenium supplement, suboptimal, thyroid autoantibody, thyroid autoimmunity, twin

## Abstract

**Objective:**

This study aimed to assess selenium status in South Korean pregnant women and its impact on maternal thyroid function and pregnancy outcomes.

**Methods:**

‘Ideal Breast Milk (IBM) Cohort Study’ included 367 pregnant women out of 442 participants and categorized into three groups based on plasma selenium levels: deficient (< 70 μg/L), suboptimal (70–99 μg/L), and optimal (≥ 100 μg/L). During the second or third trimester, various blood parameters, including selenium, thyroid-stimulating hormone, free T4, free T3, and anti-thyroid peroxidase antibody levels, were measured. Thyroid parenchymal echogenicity was assessed as another surrogate marker for thyroid autoimmunity using ultrasonography.

**Results:**

The median plasma selenium was 98.8 (range: 46.7–206.4) μg/L, and 30 individuals (8%) were categorized as deficient, while 164 (45%) were classified in the suboptimal group. Selenium deficiency was associated with markers of autoimmune thyroiditis, including positive anti-thyroid peroxidase antibody results (13.3 (deficient) vs 4.6 (optimal) %, *P* = 0.031) and thyroid parenchymal heterogeneity on ultrasound (33.3 (deficient) vs 14.6 (suboptimal) vs 17.3 (optimal) %, *P* = 0.042), independently of gestational age. The incidence of severe preeclampsia was higher in the group not taking selenium supplements, particularly among those with twin pregnancies, compared to the group taking selenium supplements (0 (selenium supplement) vs 9.0 (no supplement) %, *P* = 0.015).

**Conclusion:**

Pregnant women experience mild selenium deficiency, which can lead to significant health issues including maternal thyroid autoimmunity and obstetrical complications during pregnancy. Guidelines for appropriate selenium intake according to the stage of pregnancy and the number of fetuses are needed.

## Introduction

Selenium is an essential micronutrient that exerts its biological actions through the formation of selenoproteins, such as glutathione peroxidase (GPx), thioredoxin reductases (TXNRD), and iodothyronine deiodinases (DIOs). These selenoproteins play a pivotal role in thyroid hormone production and metabolism (1, 2, 3). First, GPx and TXNRDs protect thyroid tissues by redox control of thyrocytes and antioxidant defense during thyroid hormone synthesis in thyrocytes and the follicular lumen (4). Second, DIOs modulate thyroid hormone activities and metabolism in various organs, including the thyroid, brain, placenta, and peripheral tissues (3). Based on this biological background, epidemiological studies have shown a correlation between selenium deficiency and the prevalence of hypothyroidism (5, 6).

During pregnancy, the thyroid undergoes various alterations, including enlargement of the thyroid gland and a concomitant increase of over 50% in the production of thyroid hormones (7). The augmented thyroid hormone production is necessary to meet the maternal and fetal requirements for thyroid hormones. Therefore, it is reasonable to hypothesize that pregnant women with selenium deficiency are susceptible to thyroid dysfunction and obstetrical complications.

Currently, selenium supplementation during pregnancy has shown contradictory results in improving thyroid function and pregnancy outcomes. Administering selenium supplements to pregnant women with selenium deficiency resulted in a decrease in thyroid autoantibody levels and improved the occurrence of postpartum thyroiditis or hypothyroidism (8, 9). In contrast, a study from the UK reported negative findings, indicating that selenium supplementation had no impact on anti-thyroid peroxidase antibody (anti-TPO Ab) and thyroid-stimulating hormone (TSH) levels in pregnant women (10). Additionally, the relationship between selenium deficiency and poor pregnancy outcomes also remains controversial. Several studies showed significant relationships between selenium deficiency and poor pregnancy outcomes such as preterm birth, miscarriage, low birth weight, and preeclampsia (11, 12, 13). However, a prospective cohort study with Norwegian pregnant women showed no association between selenium and preeclampsia or pregnancy-induced hypertension (14).

This study aimed to investigate the effects of selenium deficiency on thyroid function and pregnancy outcomes using a prospective hospital-based pregnancy cohort, known as the Ideal Breast Milk (IBM) cohort (15, 16). The IBM cohort consists of pregnant women and their paired offspring, and its purpose is to investigate the effects of maternal nutrient status during pregnancy and lactating periods on obstetrical outcomes as well as fetal and infantile health. It is noteworthy that more than 30% of participants were involved in twin pregnancies, which allows for a robust twin study to be conducted.

## Materials and methods

### Study population

A secondary research study was conducted within the prospective IBM cohort, which comprised 442 pregnant women in their 2nd or 3rd trimester. These participants were recruited from Seoul National University Hospital between June 2016 and December 2019, and their offspring were subsequently enrolled in the study. The inclusion and exclusion criteria, follow-up protocol, and data collection of the IBM cohort were described previously (16). Briefly, the participants underwent three visits: 2nd or 3rd trimester, post partum 3–4 weeks, and post partum 12–15 months. Maternal urine, blood, and a 3-day diet diary were collected at the 1st and 2nd visits, and obstetrical outcomes and neonatal urine, blood, birthweight, and height were obtained at delivery. Thyroid ultrasound data of mothers were evaluated at every visit. The study was performed in accordance with the Helsinki Declaration and was approved by the Institutional Review Board of the Seoul National University Hospital (IRB No. 1512-039-727 for mothers, 1606-117-772 for children), and all mother–child pair participants provided written informed consent.

**Figure 1:**
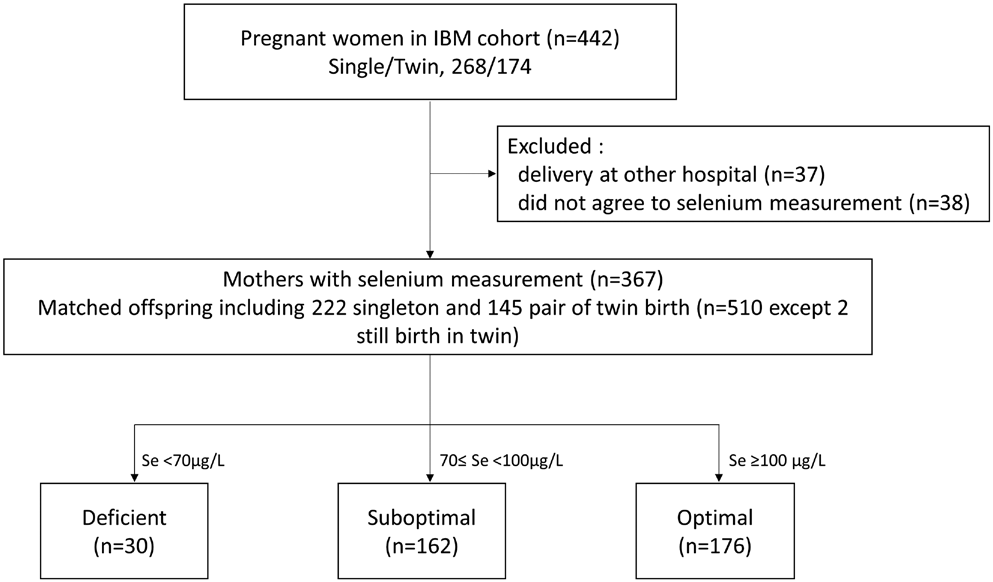
Flowchart of study participants.

Of the 442 participants in the IBM cohort, 37 women who delivered at other hospitals and 38 women who did not measure selenium were excluded. Finally, 367 mothers and 510 neonates were included in this study (Fig. 1). The clinical characteristics of the 367 maternal participants were similar to the entire IBM cohort (*n* = 442, Supplementary Table 1, see section on supplementary materials given at the end of this article).

### Measurement of plasma selenium

Maternal plasma selenium level was measured using an Inductively Coupled Plasma Mass Spectrometer (ICP-MS 7800, Agilent Technologies, Tokyo, Japan). The detection limit was 2.24 μg/L and the intra-day coefficient of variance (% CV) was 9.7 (low), 7.8 (medium), and 3.2 (high) and inter-day CV was 8.3 (low), 7.5 (medium), and 3.7 (high), respectively. According to plasma selenium concentrations, participants were divided into three groups: deficient, < 70 μg/L; suboptimal, 70–99 μg/L; and optimal, ≥ 100 μg/L. The range of the deficient group was defined by previous studies (17, 18, 19), and the optimal level was set to the concentration achieving maximal activity of glutathione peroxidases (20, 21).

### Biochemical and clinical parameters

Maternal serum TSH, free T4, and free T3 were measured using immunoradiometric assays (TSH and free T4, RIAKEY Shin Jin Medics, Seoul, Korea; free T3 and anti-TPO Ab, BRAHMS, Germany). The reference range, detection limit, intra-assay, and inter-assay CV were as follows: TSH (reference range: 0.30–5.00 mIU/L; detection limit: 0.02 mIU/L; intra-assay CV (%): 5.60/4.84/6.12; inter-assay CV (%): 7.48/4.23/5.74), free T4 (reference range: 0.70–1.80 ng/dL; detection limit: 0.04 ng/dL; intra-assay CV (%): 3.16/5.02/2.46; inter-assay CV (%): 2.73/4.88/2.77), free T3 (reference range: 0.23–0.53 ng/dL; detection limit: 0.045 ng/dL (analytical), 0.078 ng/dL (functional); intra-assay CV (%): 5.16/5.30/6.46; inter-assay CV (%): 7.90/9.50/9.20), anti-TPO Ab (reference range: < 67.3; detection limit: 22; reproducibility CV (%): 8.50/4.60; repeatability CV (%): 6.80/5.60/6.40/4.60/5.10). Urine iodine concentration (UIC) was measured by the 7900 ICP-MS apparatus (Agilent Technologies,USA). Thyroid parenchymal heterogeneity was examined by two experienced endocrinologists. Total caloric and iodine intakes were estimated by a nutritionist based on each mother’s 3-day diet diary. Gestational hypertension was defined as the occurrence of newly diagnosed hypertension in the absence of proteinuria during pregnancy, with subsequent normalization of blood pressure levels within 3 months following delivery. Preeclampsia was defined as new-onset hypertension with proteinuria (≥ 300 mg/24 hours) after 20 weeks gestation. Severe preeclampsia was defined as the concurrent manifestation of hypertension, proteinuria, and edema. Birth weight and height of neonates were converted into Z-scores considering preterm births in this study population (22).

### Statistical analysis

The normally distributed continuous variables were described as mean ± s.d. and analyzed by either Student’s *t*-test or one-way ANOVA to compare between two or three groups. Nonnormally distributed values were presented as the median (interquartile range, 25–75%), and either Mann–Whitney *U* test or Kruskall–Wallis test was used to compare between two or three groups. The categorical values were depicted as frequency (percentage) and examined by Chi-square test or Fisher’s exact test. The statistical analyses were performed using SPSS version 26.0 for Windows (SPSS Inc., USA).

## Results

### Maternal clinical characteristics and plasma selenium levels during the second and third trimesters

Table 1 demonstrated the clinical characteristics of the total participants and three categorized groups according to the plasma selenium levels. Of 367 pregnant women, the mean age was 36 ± 3 years and pre-pregnancy BMI was 22.1 ± 3.1 kg/m^2^. The primiparity was 269 (73.3%) and twin pregnancy was 145 (39.5%). Blood samples were collected during the 2nd (38.1%) and 3rd (61.9%) trimesters of pregnancy. Among all, 39 participants (10.6%) had a previous history of thyroid disease, and none of them exhibited overt thyroid dysfunction. Two patients with Graves’ disease and 29 patients with hypothyroidism were maintaining medication. Additionally, 145 participants (39.5%) took selenium supplementation through a multivitamin complex.

**Table 1:** Maternal clinical characteristics and nutritional status according to the selenium status. Data are presented as mean ± s.d. or as *n* (%).

	Total	Selenium status			*P*
		Deficient (< 70 μg/L)	Suboptimal (70–99 μg/L)	Optimal (≥ 100 μg/L)	
*n*	367	30	164	173	
Plasma selenium, μg/L	99.9 ± 23.9	62.6 ± 5.5	85.6 ± 8.3	119.4 ± 17.8	< 0.001
Clinical characteristics					
Maternal age, years	36 ± 3	37 ± 3	35 ± 3	36 ± 4	0.160
BMI, pre-pregnancy, kg/m^2^	22.1 ± 3.1	21.5 ± 3.1	22.2 ± 3.2	22.2 ± 3.3	0.495
Primiparity	269 (73.3)	21 (70.0)	117 (71.3)	131 (75.7)	0.928
Twin pregnancy	145 (39.5)	16 (53.3)	77 (47.0)	52 (30.1)	0.001
Gestational age at enrollment					0.020
2nd trimester	140 (38.1)	9 (30.0)	52 (31.7)	79 (45.7)	
3rd trimester	227 (61.9)	21 (70.0)	112 (68.3)	94 (54.3)	
T4 treatment during pregnancy	34 (9.3)	0	18 (11.0)	16 (9.2)	0.136
Previous history					
Obstetrical complication	6 (1.6)	0	1 (0.6)	5 (2.9)	0.312
Thyroid disease	39 (10.6)	1 (3.3)	20 (12.2)	18 (10.4)	0.746
Hypertensive disease	5 (1.4)	0 (0.0)	1 (0.6)	4 (2.3)	0.592
Diabetes mellitus	7 (1.9)	0 (0.0)	2 (1.2)	5 (2.9)	0.590
Family history of thyroid disease	13 (3.5)	1 (3.3)	5 (3.0)	7 (4.0)	0.909
Nutritional status					
Total caloric intake, kcal	1885 ± 435	1948 ± 445	1887 ± 429	1878 ± 439	0.772
Se supplementation	145 (39.5)	9 (30.0)	52 (31.7)	84 (48.6)	0.003
Se dose per user, μg/day	77 ± 44	63 ± 41	78 ± 51	78 ± 39	0.596

**Figure 2:**
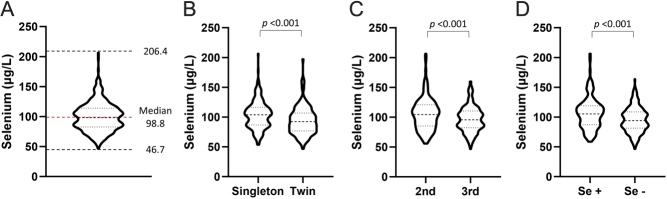
Plasma selenium levels in late pregnancy*.* (A) All participants, (B) singleton vs twin pregnancy, (C) sampling times, 2nd vs 3rd trimester of pregnancy, and (D) with (Se+) vs without (Se−) selenium supplement.

The median plasma selenium level of all participants was 98.8 μg/L and ranged from 46.7 to 206.4 μg/L (Fig. 2A). In addition, the median level of plasma selenium was significantly higher in singleton compared to twin pregnancies (103.9 vs 92.5 μg/L, *P* < 0.001, Fig. 2B), in the 2nd compared to the 3rd trimester (104.6 vs 95.6 μg/L, *P* = 0.002, Fig. 2C), and in the use of selenium supplementation (105.4 μg/L vs 94.2 μg/L, *P* < 0.001, Fig. 2D).

Among all participants, 173 (47%) were classified as having optimal selenium levels, 164 (45%) as having suboptimal levels, and 30 (8%) as deficient. The mean plasma selenium levels for these groups were 119.4 ± 17.8 μg/L, 85.6 ± 8.3 μg/L, and 62.6 ± 5.5 μg/L, respectively (Table 1). There were no significant differences in age, BMI, parity, or other medical history among the three groups (Table 1). However, there were notable differences in the ratio of singleton versus twin pregnancies and the sampling time between the 2nd and 3rd trimesters between groups (Table 1). Nutritional status-wise, the total caloric intake was similar among the three groups. However, as expected, a higher number of participants in the optimal group took selenium-containing supplements compared to the other two groups (48.6 (optimal) vs 31.7 (suboptimal) vs 30.0 (deficient) %, *P* = 0.003, Table 1). The dose of supplement among selenium-supplement users was similar between groups (78 ± 39 (optimal) vs 78 ± 51 (suboptimal) vs 63 ± 41 (deficient) μg/day, *P* = 0.596, Table 1).

### Serum thyroid hormone levels in pregnancy according to the selenium status

To explore the relationship between maternal thyroid status and plasma selenium levels, we assessed thyroid function using serum thyroid hormone levels, including TSH, free T4, and free T3. Supplementary Figs 1A, B, and C illustrate the correlations between maternal thyroid hormone levels and plasma selenium levels. TSH values ranged from 0.05 to 5.82 mIU/L, with 13 participants displaying TSH values below 0.05 mIU/L and 7 participants above 4 mIU/L (Supplementary Fig. 1A). Additionally, one participant exhibited lower free T4 levels (Supplementary Fig. 1B), and another participant had lower free T3 levels (Supplementary Fig. 1C) than the normal reference values. However, none of the participants had overt thyroid dysfunction, and there were no differences in serum levels of TSH, free T4, and free T3 among the groups based on selenium status (Table 2). Subgroup analyses of participants enrolled at different gestational ages revealed that both the 2nd and 3rd trimester groups showed no significant differences in thyroid function based on selenium status (Supplementary Table 2).

**Table 2:** Maternal thyroid-related factors according to the selenium status. Data are presented as mean ± S.D. or as *n* (%).

	Selenium status			^1^*P*	^2^*P*	^3^*P*
	Deficient (Se < 70 μg/L)	Suboptimal (Se 70–99 μg/L)	Optimal (Se ≥ 100 μg/L)			
*n*	30	164	173			
Serum TSH, mIU/L	1.26 ± 0.68	1.37 ± 0.79	1.49 ± 0.93	0.693	0.101	0.440
Serum free T4, ng/dL	0.92 ± 0.11	0.92 ± 0.14	0.91 ± 0.14	0.749	0.658	0.889
Serum free T3, ng/dL	0.29 ± 0.07	0..29 ± 0.07	0.28 ± 0.07	0.093	0.407	0.915
Anti-TPO Ab positivity	4 (13.3)	7 (4.3)	8 (4.6)	0.113	0.031	0.073
Parenchymal heterogeneity on USG	10 (33.3)	24 (14.6)	30 (17.3)	0.049	0.042	0.013

^1^*P*-value was calculated by one-way ANOVA between three groups; ^2^*P*-value was calculated by Chi-square test between deficiency and optimal groups; ^3^*P*-value was calculated by Chi-square test between deficiency and suboptimal groups.

### Indicators for autoimmune thyroiditis according to selenium status

Since lower plasma selenium levels were noted in patients with autoimmune thyroid disease (AITD, 23, 24), we evaluated thyroid autoantibody and thyroid parenchymal echogenicity. First, a total of 19 (5.2%) participants showed positive anti-TPO Ab, and the deficient group showed a higher rate of anti-TPO Ab positivity than the optimal group (13.3% vs 4.6%, *P* = 0.031, Table 2).

Secondly, we assessed thyroid gland parenchymal echogenicity using thyroid ultrasonography as a surrogate marker for AITDs, which typically display diffuse and heterogeneous echogenicity in thyroid parenchyma. Among all, 64 (17.4%) showed diffusely heterogeneous parenchymal echogenicity. The deficient group had a higher ratio of diffusely heterogeneous parenchymal echogenicity compared to the suboptimal and optimal groups (33.3% (deficient) vs 14.6% (suboptimal) vs 17.3% (optimal), *P* = 0.049, Table 2). Furthermore, we conducted subgroup analyses of participants enrolled at different gestational ages. The incidence of parenchymal heterogeneity was significantly higher in the deficient group than in other groups (38.1% (deficient) vs 14.3% (suboptimal) vs 22.3% (optimal), *P* = 0.037) in participants at the 3rd trimester (Supplementary Table 2).

Furthermore, multivariate regression analyses revealed that maternal serum selenium deficiency was a significant risk factor for the presence of anti-TPO antibodies (OR: 4.2, 95% CI: 1.0–16.7, *P* = 0.044, Supplementary Table 3), but not for parenchymal echogenicity on thyroid ultrasonography (OR: 2.1, 95% CI: 0.9–5.3, *P* = 0.103, Supplementary Table 3). This association persisted even after adjusting for relevant factors such as maternal age, pre-pregnancy BMI, gestational age at enrollment, number of fetuses, previous history of thyroid disease, maternal selenium status, and the presence of selenium supplements during pregnancy. Taken together, although participants in the selenium-deficient group did not have overt autoimmune thyroid disease or thyroid dysfunction, they showed subclinical features of autoimmune thyroiditis such as positive anti-TPO antibodies and/or heterogeneous parenchymal echogenicity on thyroid ultrasound. This was consistent across different gestational ages at enrollment, whether in the 2nd or 3rd trimester.

### Obstetrical outcomes according to the selenium status

Finally, the obstetrical outcomes were explored according to the selenium status in singleton and twin pregnancies. Supplementary Table 4 shows the maternal clinical characteristics compared between singleton and twin pregnancies. In twin pregnancies, maternal age was younger (36 ± 4 vs 35 ± 3 years, *P* = 0.027), the primiparity rate was higher (85.5% vs 65.3%, *P* < 0.001), and the proportion of sampling during the 2nd trimester was higher (46.2% vs 32.9%, *P* = 0.013) compared to singleton pregnancies. As a result, there were no differences in the rates of pregnancy-related hypertensive diseases (PRH), gestational diabetes, preterm birth, or primary cesarean sections among the three groups categorized by selenium levels, for both singleton and twin pregnancies (Table 3).

**Table 3:** Obstetrical and birth outcomes according to the selenium status in singleton and twin pregnancies. Data are presented as mean ± s.d. or as *n* (%).

	Singleton (*n* = 222)				Twin (*n* = 145)			
	Deficiency (Se < 70 μg/L)	Suboptimal (Se 70–99 μg/L)	Optimal (Se ≥ 100 μg/L)	*P*	Deficiency (Se < 70 μg/L)	Suboptimal (Se 70–99 μg/L)	Optimal (Se ≥ 100 μg/L)	*P*
Number of mothers	14 (6.3)	87 (39.2)	121 (54.5)		16 (11.0)	77 (53.1)	52 (35.9)	
Plasma selenium, μg/L	64.0 ± 5.3	87.1 ± 8.0	120.2 ± 17.3	< 0.001	61.3 ± 5.5	84.7 ± 9.0	118.7 ± 18.8	< 0.001
Clinical characteristics								
Maternal age, years	37 ± 3	36 ± 3	36 ± 4	0.393	36 ± 3	35 ± 3	35 ± 3	0.267
BMI, pre-pregnancy, kg/m^2^	21.0 ± 2.7	22.1 ± 3.1	22.0 ± 3.3	0.494	22.2 ± 3.4	22.4 ± 3.3	22.7 ± 3.3	0.828
Gestational age at enrollment				0.030				0.114
2nd trimester	3 (21.4)	21 (24.1)	49 (40.5)		6 (37.5)	31 (40.3)	30 (57.7)	
3rd trimester	11 (78.6)	66 (75.9)	72 (59.5)		10 (62.5)	46 (59.7)	22 (42.3)	
Primiparity	6 (42.9)	55 (63.2)	85 (70.2)	0.149	15 (93.8)	63 (81.8)	45 (86.5)	0.610
T4 treatment during pregnancy	0	14 (16.1)	10 (8.3)	0.081	0	4 (5.2)	7 (13.5)	0.164
Nutritional characteristics								
Total caloric intake, kcal	1690 ± 304	1884 ± 415	1906 ± 440	0.307	2120 ± 448	1889 ± 450	1811 ± 432	0.067
Supplementary selenium dose, μg/day	81 ± 59	69 ± 38	80 ± 41	0.559	61 ± 5	85 ± 9	119 ± 19	0.051
Maternal thyroid status								
Anti-TPO Ab positivity	2 (14.3)	5 (5.7)	6 (5.0)	0.378	2 (12.5)	2 (2.6)	2 (3.8)	0.232
Parenchymal heterogeneity on USG	5 (35.7)	12 (13.8)	22 (18.2)	0.131	5 (31.3)	12 (15.6)	8 (15.4)	0.290
Obstetrical outcome								
Number of mothers	14 (6.3)	87 (39.2)	121 (54.5)		16 (11.0)	77 (53.1)	52 (35.9)	
Gestational hypertension^1^	0	1 (1.1)	3 (2.5)	0.724	0	0	2 (3.8)	0.336
Preeclampsia^2^	0	0	0		0	4 (5.2)	1 (1.9)	0.805
Severe preeclampsia^3^	1 (7.1)	2 (2.3)	3 (2.5)	0.383	0	3 (3.9)	4 (7.7)	0.520
Gestational diabetes	2 (14.3)	9 (10.3)	12 (9.9)	0.864	2 (12.5)	10 (13.0)	9 (17.3)	0.808
Preterm birth	1 (7.1)	2 (2.3)	9 (7.4)	0.281	9 (56.3)	46 (59.7)	32 (61.5)	0.949
Primary cesarean section	1 (7.1)	10 (11.5)	17 (14.0)	0.662	8 (50.0)	31 (40.3)	22 (42.3)	0.809
Birth outcomes								
Number of fetus	14	87	121		32	152	104	
Height *Z*-score	−0.1 ± 0.6	−0.2 ± 0.7	−0.2 ± 0.6	0.770	−0.4 ± 0.6	−0.5 ± 0.8	−0.5 ± 0.8	0.689
Weight *Z*-score	−0.3 ± 0.9	−0.2 ± 0.1	−0.3 ± 0.8	0.829	−0.8 ± 0.8	−0.8 ± 0.7	−1.0 ± 0.8	0.666
Birthweight < 10%	1 (7.1)	7 (8.0)	8 (6.6)	0.890	7 (21.9)	28 (18.4)	24 (23.1)	0.741

^1^New-onset hypertension after 20 weeks’ gestation without proteinuria that disappears within 3 months after delivery. The definition of hypertension was either systolic blood pressure over 140 mm Hg or diastolic blood pressure over 90 mm Hg; ^2^New-onset Hypertension with proteinuria (≥ 300 mg / 24 hours) after 20 weeks’ gestation; ^3^Combination of hypertension, proteinuria, and edema after 20 weeks’ gestation.

Se, selenium; TPO Ab, thyroid peroxidase antibody; USG, ultrasonography

Furthermore, multivariate regression analysis was conducted for each obstetrical outcome using clinically relevant factors such as maternal age, pre-pregnancy BMI, number of fetuses, parity, previous history of diabetes, hypertension, gestational diabetes, gestational complications, and maternal selenium status (Supplementary Table 5). The analysis revealed that established clinical risk factors were significantly associated with each obstetrical outcome, whereas maternal selenium status did not show significant associations (Supplementary Table 5).

Interestingly, a significantly higher percentage of subjects in twin pregnancies reported supplementary selenium intake compared to singleton pregnancies (45.5% vs 35.6%, *P* = 0.032), while the fraction of the optimal group was significantly lower in twin pregnancies compared to singleton pregnancies (35.9% vs 54.5%, *P* = 0.002, Supplementary Table 4). Additionally, a higher rate of twin pregnancies underwent assisted reproductive technique (ART) procedures than singleton pregnancies (89.7 vs 30.2 %, *P* < 0.001, Supplementary Table 4). However, the rates of selenium supplementation according to ART were not different (natural pregnancy vs ART, 57.9% vs 63.3%, *P* = 0.290). Thus, the obstetrical outcomes according to the selenium supplementation in both singleton and twin pregnancies were compared without considering the history of ART. As expected, the plasma level of selenium was significantly higher in pregnant women with selenium supplementation with singleton (111.8 ± 26.4 vs 99.1 ± 20.3 μg/L, *P* < 0.001) and twin (100.1 ± 25.3 vs 88.9 ± 20.7 μg/L, *P* = 0.004) pregnancies (Table 4). Baseline clinical characteristics including maternal age, parity, gestational age at enrollment, and history of T4 treatment during pregnancy showed no difference according to the selenium supplementation in singleton and twin pregnancies (Table 4). However, pre-pregnancy BMI was significantly higher in the no-supplement group than the supplement group, but only in singleton pregnancy and not in twin pregnancy (Table 4). As for obstetrical outcomes, the rates of severe preeclampsia were significantly higher in the no-supplement group than the supplement group, in twin (0 (selenium supplement) vs 9.0 (no supplement) %, *P* = 0.015) but not singleton pregnancies (0 (selenium supplement) vs 4.2 (No supplement) %, *P* = 0.091) (Table 4). Otherwise, all other obstetrical outcomes, such as gestational diabetes mellitus, preterm birth, or primary cesarean operation, were not different (Table 4). In terms of birth outcomes, the *Z*-score of birth heights and weights showed no difference between groups (Table 4).

**Table 4:** Clinical characteristics and obstetrical outcomes according to selenium supplementation in singleton versus twin pregnancies.

	Singleton Se supplementation			Twin Se supplementation		
	No	Yes	*P*	No	Yes	*P*
Number of mothers	143 (64.4)	79 (35.6)		78 (54.2)	66 (45.8)	
Plasma selenium, μg/L	99.1 ± 20.3	111.8 ± 26.4	< 0.001	88.9 ± 20.7	100.1 ± 25.3	0.004
Clinical characteristics						
Maternal age, years	36 ± 4	36 ± 4	0.716	35 ± 3	35 ± 3	0.385
BMI, pre-pregnancy, kg/m^2^	22.3 ± 3.4	21.3 ± 2.7	0.024	22.4 ± 3.7	22.5 ± 2.8	0.815
Gestational age at enrollment			0.532			0.161
2nd trimester	49 (34.3)	24 (30.4)		40 (51.3)	27 (40.9)	
3rd trimester	93 (65.0)	55 (69.6)		38 (48.7)	41 (62.1)	
Primiparity	90 (62.9)	55 (69.6)	0.294	68 (87.2)	56 (84.8)	0.433
T4 treatment during pregnancy	19 (13.3)	4 (5.1)	0.052	4 (5.1)	7 (10.6)	0.218
Nutritional characteristics						
Total caloric intake, kcal	1851 ± 390	1947 ± 474	0.140	1887 ± 455	1889 ± 445	0.976
Supplementary selenium dose, μg/day		77 ± 41			77 ± 47	
Maternal thyroid status						
Anti-TPO Ab positivity	7 (4.9)	6 (7.6)	0.385	2 (2.6)	4 (6.1)	0.411
Parenchymal heterogeneity on USG	23 (16.1)	16 (20.3)	0.434	12 (15.4)	13 (19.7)	0.516
Obstetrical outcome						
Gestational hypertension^1^	2 (1.4)	2 (2.5)	0.618	0	2 (3.0)	0.215
Preeclampsia^2^	0	0		3 (3.8)	1 (1.5)	0.623
Severe preeclampsia^3^	6 (4.2)	0	0.091	7 (9.0)	0	0.015
Gestational diabetes	16 (11.2)	7 (8.9)	0.574	12 (15.4)	9 (13.6)	0.712
Preterm birth, n (%)	9 (6.3)	3 (3.8)	0.424	45 (57.7)	42 (63.6)	0.617
Primary cesarean section	19 (13.3)	8 (10.1)	0.479	32 (41.0)	29 (43.9)	0.843
Birth outcomes						
Number of fetus	143	79		154	132	
Height *Z*-score	−0.21 ± 0.68	−0.19 ± 0.57	0.829	−0.52 ± 0.77	−0.43 ± 0.72	0.307
Weight *Z*-score	−0.28 ± 0.85	−0.24 ± 0.82	0.677	−0.93 ± 0.80	−0.91 ± 0.72	0.832
Birthweight < 10 %, n (%)	10 (7.0)	6 (7.6)	0.658	35 (22.7)	24 (18.2)	0.329

^1^New-onset hypertension after 20 weeks’ gestation without proteinuria that disappears within 3 months after delivery. The definition of hypertension was either systolic blood pressure over 140 mm Hg or diastolic blood pressure over 90 mm Hg; ^2^New-onset hypertension with proteinuria (≥ 300 mg/24 h) after 20 weeks’ gestation; ^3^Combination of hypertension, proteinuria, and edema after 20 weeks’ gestation.

Se, selenium; BMI, body mass index; TPO Ab, thyroid peroxidase antibody; USG, ultrasonography.

## Discussion

Over 50% of the participants had suboptimal levels of plasma selenium, with 8% of them showing less than 70 μg/L. Pregnant participants in their 2nd trimester, those with singleton pregnancies, or those taking selenium supplements showed a higher proportion of optimal selenium levels (*P* < 0.001 or *P* = 0.002). Maternal selenium deficiency was associated with a higher incidence of positive anti-TPO Ab and parenchymal heterogeneity on thyroid ultrasound, while serum thyroid hormone levels showed no significant association. Notably, the incidence of severe preeclampsia was higher in the group not taking selenium supplements compared to the group taking selenium supplements, especially in cases of twin pregnancies.

The mean plasma selenium level was 99.9 ± 23.9 μg/L, which was found to be higher than the levels observed in European countries but lower than those in selenium-sufficient regions, such as the USA, Canada, and Japan (25).

While selenium replacement reduced inflammation of the thyroid in non-pregnant populations (26, 27, 28, 29), inconsistent results have been reported in pregnant women. A prospective randomized trial in 2007 showed improved anti-TPO Ab titer and thyroid echogenicity after 200 μg per day of selenium supplementation in 77 pregnant women with positive anti-TPO Ab in the 1st trimester (8). In contrast, the ‘SERENA study’ in 2019 with 45 pregnant women in the 1st trimester showed no significant difference in thyroid echogenicity, although anti-TPO Ab titer decreased after selenium replacement (9). Unlike the present study, which covered various gestational ages, the previous studies were conducted solely in the 1st trimester. In our study, the selenium-deficient group consistently exhibited subclinical features of autoimmune thyroiditis across different gestational ages, including the 2nd and 3rd trimesters. The anti-inflammatory capacity of selenium may elucidate its effects on thyroid autoimmunity.

Based on a previous study highlighting the negative impact of selenium deficiency on pregnancy outcomes, selenium supplementation has been recommended in selenium-deficient areas (30). In the present study, we observed a significantly higher incidence of severe preeclampsia in the group not receiving selenium supplementation among twin pregnancies. Meanwhile, in contrast to the stratified analyses, the multivariate analysis, in combination with established clinical risk factors, showed no statistically significant increasing tendency for severe preeclampsia in the selenium non-supplement group. This suggests that one of the covariates, these implied clinical factors, was a strong confounder in the association between selenium and severe preeclampsia. Notably, established clinical risk factors for preeclampsia hold more predictive power than selenium status in multivariate analysis. However, the health impact of nutrient factors remains significant, even if their effect size is not as robust as that of established clinical factors, as it can be easily corrected through dietary modifications. In contrast to our findings, several investigations conducted in areas where selenium levels remain within the optimal range have yielded contradictory results concerning preeclampsia (10, 31, 32). Norwegian pregnant women exhibited suboptimal plasma selenium levels, similar to our study (14). Nevertheless, their results diverged from ours, as they detected no discernible correlation between selenium intake and preeclampsia. This disparity may be attributed to the incorporation of twin pregnancies in our study and our particular emphasis on severe preeclampsia. As the present study was conducted in a tertiary referral center of the hospital and the prevalence of twin pregnancies has increased due to the rising utilization of ART including IVF (33, 34), this study had a high incidence of twin pregnancies. Indeed, the potential association between IVF and the incidence of preeclampsia has been established (35).

Recently, a study from the Odense Child Cohort suggested an association between lower serum selenium and selenoprotein P levels during pregnancy, which were significantly linked to gestational diabetes (36). However, our present study revealed no significant association between selenium levels and gestational diabetes. One explanation for this inconsistency could be the variance in baseline clinical characteristics, particularly the risk factors for gestational diabetes, between the Danish cohort and our Asian study population. Further investigation involving high-risk patients is warranted to validate these findings, utilizing a larger study cohort with diverse risk factors and conducting detailed analyses of selenoproteins.

This observational cohort study has raised several questions. First, plasma selenium levels were significantly lower in the 3rd trimester compared to the 2nd trimester and in twin pregnancies compared to singleton pregnancies. The proportion of twin pregnancies was higher in participants who were recruited during the 2nd trimester. During pregnancy, both these factors undergo an increase in blood volume, which can dilute trace elements in the blood. Therefore, the primary question arose as to whether these diluted plasma selenium levels in the 3rd trimester or twin pregnancies have any health impacts. Indeed, standards for adequate selenium levels have been established separately for each trimester (37, 38). Although the present study is an observational cohort study, it demonstrates that plasma selenium levels decrease during pregnancy, with adverse health impacts on thyroid autoimmunity. Therefore, it is crucial to closely monitor selenium levels in the 3rd trimester. Additionally, there is a need for more tailored cut-off values for selenium that may predispose individuals to thyroid autoimmunity, with consideration for different trimesters of pregnancy.

The number of fetuses is another pivotal factor in determining plasma selenium levels. Notably, even though more twin pregnancies took selenium supplements than singleton pregnancies, the plasma selenium level was significantly lower in twin pregnancies than in singleton pregnancies. When we analyzed the plasma selenium levels of participants who took selenium supplements and those who did not, both singleton and twin pregnancies had about a 12% increase in plasma selenium levels, even though they took similar daily amounts of selenium supplements. However, severe preeclampsia occurred only in those who did not take selenium supplements in singleton and twin pregnancies, and the event rate was higher in twin pregnancies. Therefore, it is reasonable to deduce that lower selenium levels may impact adverse pregnancy complications, particularly severe preeclampsia, especially in susceptible pregnancies such as twins. These results also suggest that the low selenium concentration in twin pregnancies is not solely a result of dilution due to increased plasma volume but is indeed associated with health risks. Subsequently, recommendations for the required daily selenium intake and optimal plasma selenium levels should be established, informed by evidence of health impacts, particularly in twin pregnancies. A further large-scale clinical study is needed to delve deeper into this matter. A randomized controlled study with a larger sample size for each singleton or twin pregnancy, and in the 2nd or 3rd trimester, is needed to investigate the impact of selenium supplements on pregnancy outcomes and maternal thyroid health.

The strength of our study lies in the considerable number of twin pregnancy cases in our cohorts and the low number of missing values in the database. However, the limitations of our study include a small sample size of the selenium-deficient group and a lack of information about compliance with selenium supplementation. Additionally, this study solely utilized serum selenium as the biomarker for selenium assessment. Given that serum concentrations of selenoprotein P and selenoprotein P-autoantibody are known to be related to deiodinase activity (39, 40), further study measuring selenoproteins is necessary to enhance the clinical implications of selenium biology.

In conclusion, pregnant women in South Korea exhibited mild selenium deficiency, which carries significant health implications during pregnancy. Selenium deficiency can increase the risk of thyroid autoimmunity among pregnant individuals. Moreover, lower plasma selenium levels, especially in the absence of selenium-containing supplements, were associated with severe preeclampsia in twin pregnancies. Therefore, careful monitoring of selenium status should be considered for late pregnancies and high-risk individuals, such as those with twin pregnancies.

## Supplementary Materials

Supplementary Material

## Declaration of interest

The authors report no conflict of interest that could be perceived as prejudicing the impartiality of the study reported.

## Funding

This work was supported by Korea Environmental Industry & Technology Institute (KEITI) through the ‘Core Technology Developmenthttp://dx.doi.org/10.13039/100006180 Project for Environmental Diseases Prevention and Management’, funded by Korea Ministry of Environmenthttp://dx.doi.org/10.13039/501100003562 (MOE) (2022003310006) and the Seoul National Universityhttp://dx.doi.org/10.13039/501100002551 Hospital Research Fund (Grant nos 30-2017-0070 and 04-2017-3010). The funding source was not involved in study design; in the collection, analysis, and interpretation of data; in the writing of the report; and in the decision to submit the article for publication.

## Author contribution statement

Study design: SWC, YJP, GJC, JKC, CHS, CWC; data acquisition: CWC, YJS, DLJ, YAL, YJP, JKJ, SWC; data analysis: CWC, SKP, KK; data interpretation: CWC, KK, SWC; drafting the article and critical review of revisions: CWC, KK, SWC; final approval to submit: CWC, KK, SWC, GJC; obtaining funding: SWC.
