# Supplementary material for: Selenium levels and their association with thyroid autoimmunity and severe preeclampsia in pregnancy: Insights from a prospective ideal breast milk cohort study
Source: Eur Thyroid J. 2024 Jul 9;13(4):e240007. doi: 10.1530/ETJ-24-0007 (PMC11301555; doi:10.1530/ETJ-24-0007)

Supplementary Table 1. Clinical and nutritional characteristics of IBM cohort

	**IBM cohort, total** (N=442)	**Stuty population** (N=367)	***P*-value**
Clinical characteristics			
Maternal age, years	36 ± 4	36 ± 3	0.957
BMI, pre-pregnancy, kg/m^2	22.1 ± 3.3	22.1 ± 3.2	0.852
Twin pregnancy, n (%)	174 (39.4)	145 (39.5)	0.904
Gestational age at enrollment, n (%)			0.870
2nd trimester	176 (39.8)	140 (38.1)	
3rd trimester	266 (60.2)	227 (61.9)	
Parity, n (%)			0.726
0	339 (76.7)	269 (73.3)	
1	79 (17.9)	76 (20.7)	
≥2	24 (5.4)	22 (6.0)	
T4 treatment during pregnancy, n (%)	40 (9.0)	34 (9.3)	0.878
Previous history, n (%)			
Obstetrical complication	7 (1.6)	6 (1.6)	0.909
Thyroid disease	48 (10.9)	39 (10.6)	0.807
Hypertensive disease	7 (1.6)	5 (1.4)	0.795
Diabetes mellitus	11 (2.5)	7 (1.9)	0.577
Family history of thyroid disease, n (%)	16 (3.6)	13 (3.5)	0.983
Nutritional characteristics			
Total caloric intake, kcal	1885 ± 435	1878 ± 433	0.796
Supplementary selenium intake, n (%)	175 (39.6)	145 (39.5)	0.914

Supplementary Table 2. Maternal thyroid function, autoantibody, and thyroid parenchymal echogenicity on sonography according to the selenium status during the 2nd and 3rd trimesters

**Selenium status**	**Deficient** (Se <70 μg/L)	**Suboptimal** (Se 70-99 μg/L)	**Optimal** (Se ≥100 μg/L)	***P*-value**
2nd trimester	N=9	N=52	N=79	
Serum TSH, mIU/L	1.30 ± 0.70	1.23 ± 0.76	1.32 ± 0.70	0.785
Serum free T4, ng/dL	0.86 ± 0.11	0.89 ± 0.13	0.91 ± 0.15	0.593
Serum free T3, ng/dL	0.25 ± 0.07	0.29 ± 0.08	0.27 ± 0.06	0.048
Anti-TPO Ab positivity, n (%)	2 (22.2)	1 (1.9)	4 (5.1)	0.079
[1]Parenchymal heterogeneity, n (%)	2 (22.2)	8 (15.4)	9 (11.4)	0.595
3rd trimester	N=21	N=112	N=94	
Serum TSH, mIU/L	1.24 ± 0.69	1.44 ± 0.80	1.62 ± 1.06	0.140
Serum free T4, ng/dL	0.95 ± 0.11	0.94 ± 0.14	0.92 ± 0.14	0.450
Serum free T3, ng/dL	0.31 ± 0.06	0.30 ± 0.07	0.29 ± 0.07	0.572
Anti-TPO Ab positivity, n (%)	2 (9.5)	6 (5.4)	4 (4.3)	0.614
[1]Parenchymal heterogeneity, n (%)	8 (38.1)	16 (14.3)	21 (22.3)	0.037

[1]Parenchymal heterogeneity was assessed using thyroid ultrasound (USG).
Se, selenium; TSH, thyroid stimulating hormone; TPO Ab, thyroid peroxidase antibody.

Supplementary Table 3. Association between maternal indicators for autoimmune thyroiditis and maternal selenium levels: multivariate regression analysis

		Anti-TPO Ab positivity		Parenchymal heterogeneity	
	N	OR (95% CI)	*P-value*	OR (95% CI)	*P-value*
Maternal serum selenium[1]					
Optimal	173	1.00		1.00	
Suboptimal	164	1.2 (0.4–3.6)	0.743	0.8 (0.4–1.5)	0.472
Deficiency	30	4.2 (1.0–16.7)	0.044	2.1 (0.9–5.3)	0.103
Maternal age					
< 35 years	142	1.00		1.00	
≥ 35 years	225	1.0 (0.4–2.8)	0.926	1.8 (1.0–3.3)	0.067
BMI (kg/m^2), pre-pregnancy					
<18.5	280	1.1 (0.2–5.2)	0.946	2.1 (0.8–5.3)	0.111
18.5-24.9	29	1.00		1.00	
≥25	58	0.4 (0.0–3.0)	0.354	1.1 (0.5–2.4)	0.823
Gestational age at enrollment					
2nd trimester	140	1.00		1.00	
3rd trimester	227	0.9 (0.3–2.5)	0.855	1.6 (0.9–2.9)	0.138
Selenium supplement					
Yes	145	1.00		1.00	
No	221	0.5 (0.2–1.3)	0.132	0.7 (0.4–1.3)	0.288
Multiple pregnancy					
No	222	1.00		1.00	
Yes	145	0.6 (0.2–1.7)	0.353	1.0 (06–1.9)	0.927
Previous history of thyroid disease[2]					
No	327	1.00		1.00	
Yes	40	1.9 (0.5–7.3)	0.334	1.3 (0.6–3.1)	0.555

Abbreviation, BMI, Body mass index; TPOAb, Thyroid peroxidase antibody

1. Maternal serum selenium is defined in each criterion, as optimal (≥ 100 μg/L), suboptimal (70–99 μg/L), deficient (< 70 μg/L)
2. Thyroid disease includes hypothyroidism and hyperthyroidism. There were no patients with previous history of thyroid cancer.

Supplementary Table 4. Multivariate analysis of pregnancy-related hypertensive diseases, gestational diabetes mellitus, preterm birth and primary cesarean section according to the maternal factors

		Pregnancy-related hypertensive diseases[3]		GDM	
	N	OR (95% CI)	*P-value*	OR (95% CI)	*P-value*
Maternal serum selenium[1]					
Optimal	173	1.00		1.00	
Suboptimal	164	0.7 (0.2–2.2)	0.562	1.1 (0.5–2.5)	0.836
Deficiency	30	0.7 (0.1–6.4)	0.715	2.7 (0.7–10.1)	0.135
Maternal age					
< 35 years	142	1.00		1.00	
≥ 35 years	225	2.2 (0.6–8.1)	0.222	0.8 (0.4–1.7)	0.539
BMI (kg/m^2), pre-pregnancy					
<18.5	280	1.3 (0.1–11.4)	0.839	0.9 (0.2–4.0)	0.838
18.5-24.9	29	1.00		1.00	
≥25	58	2.1 (0.6–7.4)	0.232	2.7 (1.1–6.6)	0.034
Multiple pregnancy					
No	222	1.00		1.00	
Yes	145	6.3 (1.4–27.6)	0.014	1.0 (0.4–2.2)	0.926
Nulliparity					
No	269	1.00		1.00	
Yes	98	9.6 (1.4–65.1)	0.021	0.5 (0.2–1.6)	0.238
Previous history of DM					
No	360	1.00		1.00	
Yes	7	15.5 (0.9–280.6)	0.064	7.8 (0.5–111.8)	0.129
Previous history of GDM					
No	363	1.00		1.00	
Yes	4	Not evaluable	0.997	23.8 (1.4–406.0)	0.029
Previous history of hypertension					
No	362	1.00		1.00	
Yes	5	Not evaluable	0.999	0.9 (0.0–543.5)	0.974
Previous history of gestational complication[2]					
No	363	1.00		1.00	
Yes	4	Not evaluable	0.999	0.4 (0.0–191.3)	0.754
		**Preterm birth[4]**		**Primary C/S[5]**	
	N	OR (95% CI)	*P-value*	OR (95% CI)	*P-value*
Maternal serum selenium[1]					
Optimal	173	1.00		1.00	
Suboptimal	164	0.7 (0.3–1.5)	0.372	1.0 (0.5–2.0)	0.992
Deficiency	30	0.4 (0.1–1.7)	0.237	1.3 (0.4–4.3)	0.632
Maternal age					
< 35 years	142	1.00		1.00	
≥ 35 years	225	1.3 (0.6–2.7)	0.559	1.3 (0.7–2.6)	0.390
BMI (kg/m^2), pre-pregnancy					
<18.5	280	0.6 (0.1–2.2)	0.421	0.5 (0.1–1.7)	0.236
18.5-24.9	29	1.00		1.00	
≥25	58	1.1 (0.4–2.8)	0.805	0.5 (0.2–1.3)	0.173
Multiple pregnancy					
No	222	1.00		1.00	
Yes	145	52.5 (17.3–159.9)	<0.001	3.5 (1.8–6.8)	<0.001

Nulliparity					
No	269	1.00		1.00	
Yes	98	0.3 (0.1–0.9)	0.031	0.2 (0.1–0.7)	0.010
Previous history of DM					
No	360	1.00		1.00	
Yes	7	9.3 (0.5–189.6)	0.147	Not evaluable	0.999
Previous history of GDM					
No	363	1.00		1.00	
Yes	4	Not evaluable	0.999	Not evaluable	0.999
Previous history of hypertension					
No	362	1.00		1.00	
Yes	5	5.3 (0.2–180.1)	0.350	Not evaluable	0.999
Previous history of gestational complication[2]					
No	363	1.00		1.00	
Yes	4	0.1 (0.0–3.1)	0.176	Not evaluable	0.999

Abbreviation, BMI, Body mass index; DM, diabetes; GDM, gestational diabetes; C/S, Cesarean section

1. Maternal serum selenium defines in each criterion, optimal (≥ 100 μg/L), suboptimal (70–99 μg/L), deficient (< 70 μg/L)

2. Previous history of gestational complication includes preterm birth and gestational hypertension.

3. Pregnancy induced hypertension includes gestational hypertension, preeclampsia, and severe preeclampsia. There was no history of hypertension and previous history of gestational diabetes and gestational complication in pregnant women with pregnancy induced hypertension.

4. There was no previous history of GDM in pregnant women with preterm birth.

5. There was no history of diabetes and hyptertension, and previous history of gestational diabetes and gestational complication in pregnant women with primary Cesarean section.

Supplementary Table 5. Clinical and nutritional characteristics according to singleton or twin pregnancy.

	**Singleton** (N=222)	**Twin** (N=145)	***P*-value**
Clinical characteristics			
Maternal age, years	36 ± 4	35 ± 3	0.027
BMI, pre-pregnancy, kg/m^2	22.0 ± 3.2	22.4 ± 3.3	0.156
2nd trimester at sampling, n (%)	73 (32.9)	67 (46.2)	0.013
Primiparity, n (%)	145 (65.3)	124 (85.5)	<0.001
T4 treatment during pregnancy, n (%)	23 (10.4)	11 (7.6)	0.374
ART*, n (%)	67 (30.2)	130 (89.7)	<0.001
Previous history, n (%)			
Obstetrical complication	5 (2.3)	1 (0.7)	0.418
Thyroid disease	28 (12.6)	11 (7.6)	0.118
Hypertensive disease	5 (2.3)	0	0.162
Diabetes mellitus	6 (2.7)	1 (0.7)	0.251
Family history of thyroid disease, n (%)	9 (4.1)	4 (2.8)	0.499
Nutritional characteristics			
Total caloric intake, kcal	1887 ± 426	1887 ± 449	0.997
Supplementary selenium intake, n (%)	79 (35.6)	66 (45.5)	0.032
Supplementary selenium amount, μg	77 ± 41	77 ± 47	0.135
Selenium-optimal group, n (%)	121 (54.5)	52 (35.9)	0.002
Thyroid status			
**Thyroid function parameters**			
Serum TSH, mIU/L	1.4 ± 0.8	1.5 ± 0.9	0.356
Serum free T4, ng/dL	0.93 ± 0.13	0.90 ± 0.15	0.041
Serum free T3, ng/dL	0.29 ± 0.07	0.28 ± 0.07	0.089
**Anti-TPO Ab positivity, n (%)**	13 (5.9)	6 (4.1)	0.450
**Parenchymal heterogeneity on USG, n (%)**	39 (17.6)	25 (17.2)	0.897

* ART during current pregnancy.

BMI, body mass index; ART, assisted reproductive technique; TSH, thyroid stimulating hormone; TPO Ab, thyroid peroxidase antibody; USG, ultrasonography.

Supplementary Figure 1. Correlations between maternal thyroid function parameters and plasma selenium concentrations. (A) TSH, (B) free T4, and (C) free T3. Blue continuous line presents trend line, and grey area shows distribution of values.

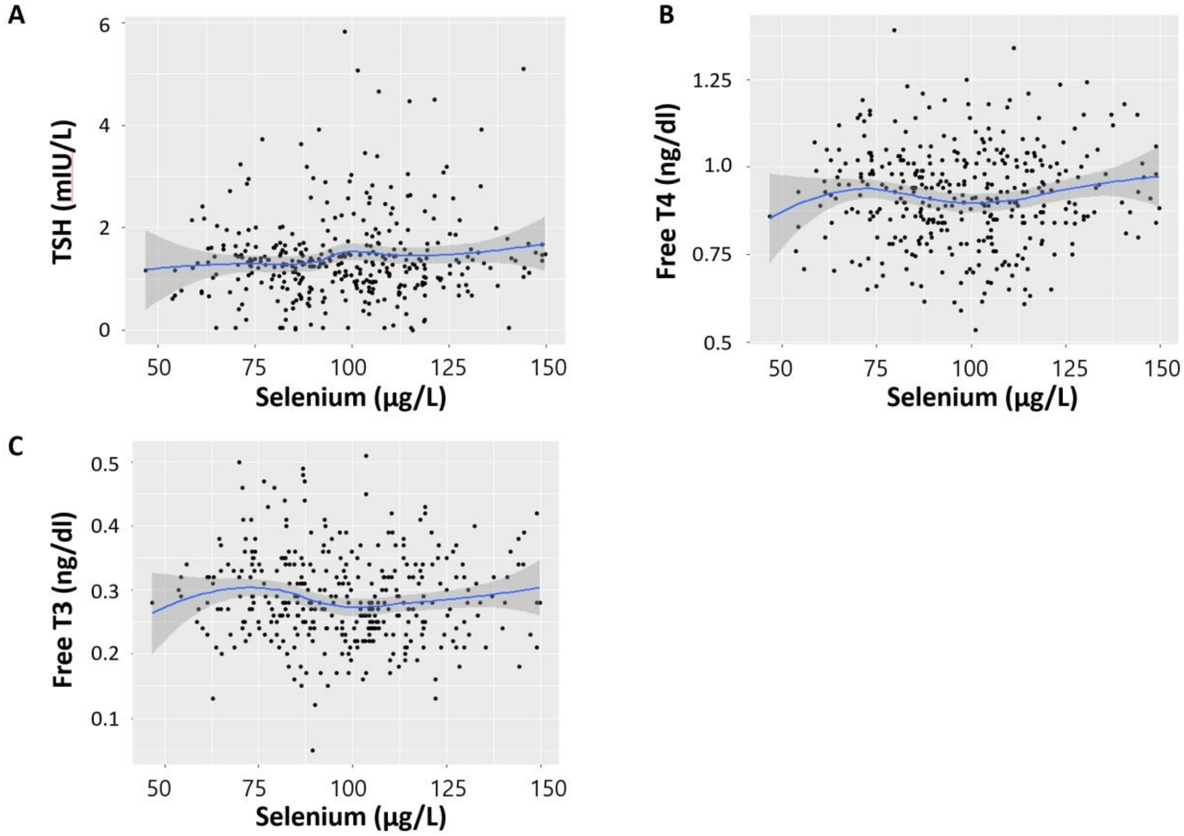

Supplement: Supplementary Material [file supplementary_material.pdf]
